# *In vitro* resensitization of multidrug-resistant clinical isolates of *Enterococcus faecium* and *E. faecalis* through phage-antibiotic synergy

**DOI:** 10.1128/aac.00740-24

**Published:** 2024-12-19

**Authors:** Pooja Ghatbale, Govind Prasad Sah, Sage Dunham, Ethan Khong, Alisha Blanc, Alisha Monsibais, Andrew Garcia, Robert T. Schooley, Ana G. Cobián Güemes, Katrine Whiteson, David T. Pride

**Affiliations:** 1 Department of Pathology, University of California 8784 , San Diego, California, USA; 2 Department of Molecular Biology and Biochemistry, University of California 8788 , Irvine, California, USA; 3 Department of Medicine, University of California 8784 , San Diego, California, USA; University of Pittsburgh School of Medicine, Pittsburgh, Pennsylvania, USA

**Keywords:** synergy, cooperativity, antibiotics, bacteriophages, resensitization

## Abstract

Bacteriophages are an increasingly attractive option for the treatment of antibiotic-resistant infections, but their efficacy is difficult to discern due to the confounding effects of antibiotics. Phages are generally delivered in conjunction with antibiotics, and thus, when patients improve, it is unclear whether the phages, antibiotics, or both are responsible. This question is particularly relevant for enterococcus infections, as limited data suggest phages might restore antibiotic efficacy against resistant strains. Enterococci can develop high-level resistance to vancomycin, a primary treatment. We assessed clinical and laboratory isolates of *Enterococcus faecium* and *Enterococcus faecalis* to determine whether we could observe synergistic interactions between phages and antibiotics. We identified synergy between multiple phages and antibiotics including linezolid, ampicillin, and vancomycin. Notably, antibiotic susceptibility did not predict synergistic interactions with phages. Vancomycin-resistant isolates (*n* = 6) were eradicated by the vancomycin-phage combination as effectively as vancomycin-susceptible isolates (*n* = 2). Transcriptome analysis revealed significant gene expression changes under antibiotic-phage conditions, especially for linezolid and vancomycin, with upregulated genes involved in nucleotide and protein biosynthesis and downregulated stress response and prophage-related genes. While our results do not conclusively determine the mechanism of the observed synergistic interactions between antibiotics and phages, they do confirm and build upon previous research that observed these synergistic interactions. Our work highlights how using phages can restore the effectiveness of vancomycin against resistant isolates. This finding provides a promising, although unexpected, strategy for moving forward with phage treatments for vancomycin-resistant *Enterococcus* infections.

## INTRODUCTION

Shortly after the introduction of penicillin and sulfonamides to clinical medicine, antibiotic resistance emerged as a significant issue (1). Over the past few decades, the prevalence of antibiotic-resistant microorganisms has risen at an alarming rate. Unfortunately, the use of currently available antibiotics promoted the emergence of pathogenic bacterial strains with reduced susceptibility (2, 3). The rapid emergence of antimicrobial resistance (AMR) is a pressing public health issue that poses a significant threat to global health and well-being. The Centers for Disease Control and Prevention (CDC) reported in 2019 that AMR organisms killed at least 1.27 million people globally and approximately 5 million fatalities were associated with AMR pathogens in some manner (3). The inappropriate use of antimicrobial drugs in humans and animals has been one of the main contributors to the rise of AMR, resulting in a growing number of infections that are difficult and sometimes impossible to treat. AMR is not only a concern for individuals but also the healthcare system and economy, as it requires costly and time-consuming measures to develop new drugs and control the spread of resistant bacteria. In the United States alone, antibiotic-resistant bacteria are responsible for 50%–60% of hospital-acquired infections (4).

Despite being commensal to the human gastrointestinal and genitourinary tracts, Gram-positive enterococci possess high levels of antimicrobial resistance. As a result, they can cause challenging infections that are associated with mortality rates ranging from 19% to 48% (5, 6). These organisms are commonly linked to urinary tract infections arising from medical instrumentation and frequent antimicrobial use. In addition, they can give rise to infections in the abdominal and pelvic areas, surgical wounds, endocarditis, bacteremia, sepsis, and, albeit rarely, meningitis in newborns (7). Some enterococci possess intrinsic resistance to commonly employed antibiotics like penicillin and ampicillin. Moreover, they demonstrate elevated resistance to most cephalosporins and all semi-synthetic penicillins due to the presence of low-affinity penicillin-binding proteins (8). Biofilm formation is an additional important pathogenic feature that may contribute to the creation of bacterial reservoirs that shield pathogens from elimination by antibiotics and increase the risk of morbidity and mortality (9, 10). *Enterococcus faecalis* and *Enterococcus faecium* are the dominant species of infective enterococci and are responsible for the vast majority of all enterococcal infections in humans (7). It is noteworthy that *E. faecium* and *E. faecalis* are ranked as the third and fourth most common causes of nosocomial infections globally (4). The first case of vancomycin-resistant enterococci (VRE) was reported in 1986 from Europe and in 1988 from the United States (11) and later in 1995, the CDC’s Healthcare Infection Control Practices Advisory Committee (HICPAC) classified VRE as a significant emerging pathogen, and recommended aggressive infection control measures to prevent its spread (12). As VRE becomes more common, the difficulty in treating enterococcal infections has significantly increased. As a result, there is an urgency to develop new therapeutic approaches (13, 14).

Bacteriophages (“phages” for short) can be used as therapeutic agents to treat multidrug-resistant bacterial infections with minimal side effects. Phages are viruses that infect specific bacterial hosts and replicate within them to produce an abundance of progeny before killing their bacterial hosts (15–18). Phages have not only been shown to be effective in controlling enterococcal growth *in vitro* but also have proven to be successful in treating infections in animal models (19–21). Likewise, enterococcal phages have been effectively used to disrupt biofilms and treat infections that are usually harder for antibiotics to penetrate (19). Furthermore, *in vivo* mice studies showed that, unlike antibiotics, phage therapy is highly specific and effective against certain bacterial species without noticeable toxicity or adverse side effects (22–24). Despite these qualities, the exclusive use of phages to treat bacterial infections suffers from the challenge of treatment-emergent phage-resistant bacterial strains (25–28) as well as phage production and commercialization challenges. Bacteria and phages continuously battle to evolve into a more resilient version of themselves and during this process, bacteria acquire/modify their defense mechanisms such as restriction-endonuclease systems, cell surface receptor alteration, CRISPR-Cas immunity, and abortive infections (29–31); thus, bacterial resistance to phages remains a significant consideration.

Phage cocktails can be used as an effective strategy to prevent the emergence of bacteria resistant to phages. In fact, phage cocktails in *in vitro* settings are highly effective in controlling the growth of antibiotic and phage-resistant bacterial strains as compared to the use of single phages (32–34). We have previously shown that cocktails of two or three phages were effective in limiting the *in vitro* growth of vancomycin-resistant *Enterococcus faecium* and *faecalis* strains that were originally resistant against some individual phages used in the cocktail (35). Furthermore, several clinical trial studies have documented successful uses of phage cocktails on a variety of bacterial pathogens (36–38). Apart from the investigations involving phage cocktails, certain studies have documented encouraging outcomes when exploring the effects of combining phages with antibiotics (33, 39). A handful of studies in recent years have demonstrated the synergistic effects of phage-antibiotic combinations on vancomycin-resistant *E. faecium* and *E. faecalis* (27, 40, 41) strains under various conditions. In the study reported here, we aimed to confirm and extend prior work to include a greater emphasis on clinical VRE isolates.

The precise molecular mechanisms underlying the enhanced killing of host cells through phage-antibiotic synergistic interactions remain unclear. It has been shown that strain susceptibility to antibiotics does not predict synergistic interactions with phages. This has been demonstrated in *Escherichia coli* (42), *Acinetobacter baumannii* (43), and *Pseudomonas aeruginosa* (44). To address this knowledge gap, we investigated how the combination of phages and antibiotics leads to the resensitization of bacterial strains to the antibiotic. Our approach involved comprehensive screening and *in vitro* testing of various phage and antibiotic combinations to identify the most effective synergistic pairs against different enterococcal strains. In addition, we examined the transcriptome of the bacterial host cells from the most successful phage-antibiotic conditions to identify key cellular pathways that contribute to synergistic effects.

## RESULTS

### Characterization of bacteria and phage isolates

Four isolates each of *E. faecium* (Tx1330, EF98PII, EF208PII, and NYU) and *E. faecalis* (DP11, EF116PII, EF140PII, and V587) were identified from patients with infections at UCSD Health or were type strains (Table 1). These isolates were chosen based on their vancomycin (VAN) susceptibility profiles. Isolates Tx1330 and DP11 were vancomycin susceptible, while EF98PII, EF208PII, NYU, EF116PII, EF140PII, and V587 were vancomycin resistant (Table 1). Some of these isolates were sequenced as part of this study; however, some had previously undergone whole-genome sequencing (35).

**TABLE 1:** Enterococcus isolates used in this study and their antibiotic susceptibility profiles, and the phages used for synergy experiments^*a*^

Enterococcus strains		GenBank accession	Vancomycin sensitivity	Antibiotic resistance	Phages used for PAS study
*E. faecium*	Tx1330	GCA_003583905.1	S	CZO, CXI, GEN, CLI, TRS	Bop
	EF98PII	SAMN39584152	R	VAN, AMP, BEN, TET	Ben
	EF208PII	SAMN36748517	R	VAN, CZO, CXI, CLI, ERY, GEN, LEV, MIN, penicillin G, STR-Syn, TET, TRS	Bop
	NYU	SAMN36748518	R	VAN, AMP, CZO, CXI, GEN, CLI, TRS, ERY, LEV, MIN, MOX, BEN, TET	Ben
*E. faecalis*	DP11	JALPNV000000000	S	GEN	ReUmp
	EF116PII	SAMN39584151	R	VAN, GEN, TET	PL
	EF140PII	SRX21185070	R	VAN, CZO, CXI, CLI, ERY, GEN, GEN-Syn, LEV, MIN, STR-Syn, TET, TRS	Bob
	V587	GCA_000394175.1	R	VAN, GEN, ERY, CZO, CXI, CLI, TRS	Ben

^*a*^ The EUCAST System for Antimicrobial Abbreviations was used to name antibiotics. VAN: vancomycin, TET: tetracycline, CZO: cefazolin, CXI: cefoxitin, CLI: clindamycin, ERY: erythromycin, GEN: gentamicin, LEV: levofloxacin, MIN: minocycline, STR-Syn: streptomycin synergy, TET: tetracycline, TRS: trimethoprim-sulfamethoxazole.

**Fig 1:**
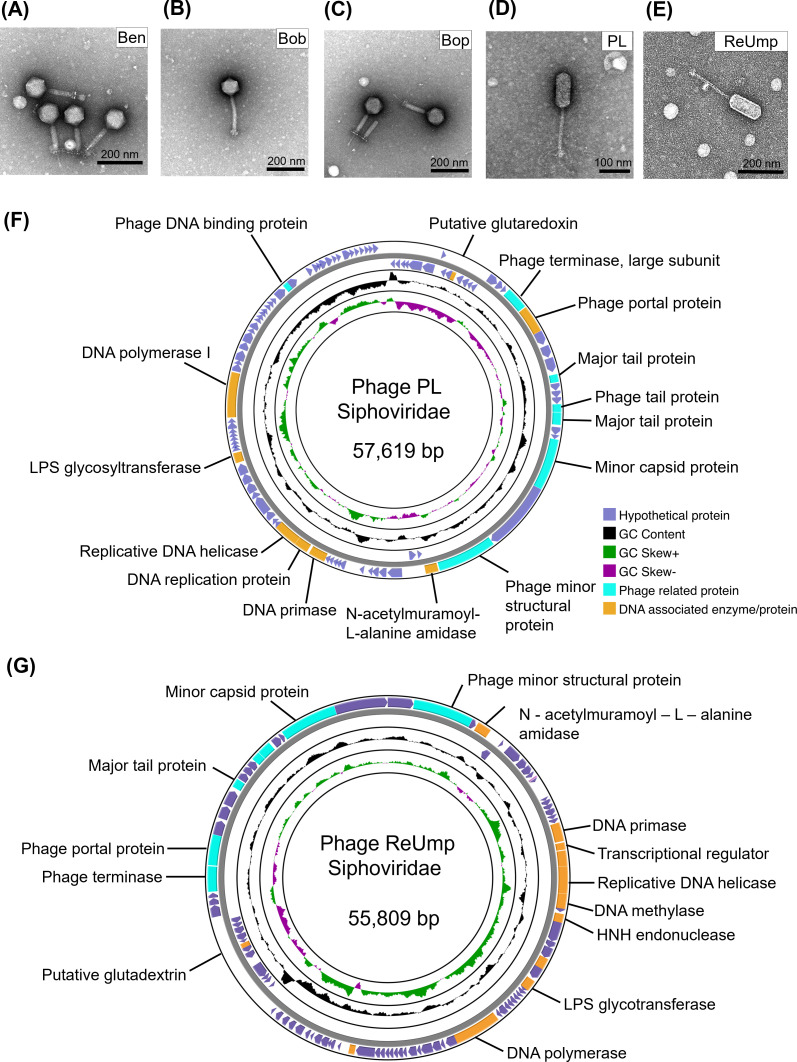
Morphological and genomic characterization of *E. faecium and E. faecalis* phages. Myovirus morphologies were observed for phages Ben, Bob, and Bop (**A–C**). Siphovirus morphologies were observed for phages PL and ReUmp (**D and E**). Genome maps of phage PL (**F**) and ReUmp (**G**). The outermost circle shows open reading frames (ORF) of predicted proteins. Phage structural proteins are highlighted in cyan; DNA-associated proteins are shown in yellow; and hypothetical proteins are shown in purple.

We also characterized phages active against many of the *E. faecium* and *E. faecalis* isolates we found. Some of these phages had previously been characterized in our prior studies (35). Phage morphologies were obtained using transmission electron microscopy (TEM; Fig. 1A through E). The TEM confirmed phage morphologies consistent with myoviruses and siphoviruses, respectively (Fig. 1). Specifically, phages Ben, Bob, and Bop displayed large icosahedral heads with medium-sized contractile tails consistent with myovirus morphologies (Fig. 1A through C), while PL and ReUmp exhibited prolate shaped heads with noncontractile tails, consistent with siphovirus morphologies (Fig. 1D and E). We obtained the phage genome sizes using whole-genome sequencing (Table 2). The newly characterized phages PL and ReUmp appeared to have lytic lifestyles since they do not encode integrases nor excisionases (Fig. 1F and G). Phages PL and ReUmp clustered with other *Enterococcus* phages (Fig. S1A). The closest relative to phage PL was *Enterococcus* phage Ef2.2, and the closest relative to phage ReUmp was *Enterococcus* phage SDS1 (Fig. S1B and C).

**TABLE 2:** Enterococcus phages used in this study^*a*^

Phage	Genome size (bp)	Morphology	Isolation source	Isolation host	Reference
Ben	151,985	Myoviridae	Orange County sewage, CA	*E. faecalis* Yi6-1	(35)
Bob	142,921	Myoviridae	Orange County sewage, CA	*E. faecalis* B3286	(35)
Bop	153,454	Myoviridae	Orange County sewage, CA	*E. faecalis* Yi6-1	(35)
PL	57,619	Siphoviridae	Point Loma sewage, CA	*E. faecalis* EF116PII	This study
ReUmp	55,809	Siphoviridae	Orange County sewage, CA	*E. faecalis* Yi6-1	This study

^*a*^ Genome sizes were obtained from whole-genome sequencing. Morphologies were obtained via TEM.

### A liquid assay to demonstrate phage-antibiotic synergy

We developed a comprehensive screening assay to determine optimum phage and antibiotic concentrations that could efficiently inhibit the growth of vancomycin-resistant enterococcus (VRE) and vancomycin-susceptible enterococcus (VSE) isolates. To perform this assay, we set up 96-well plates with increasing concentrations of antibiotics across the x-axis and increasing concentrations of the phages across the y-axis (Fig. S2). We examined three different antibiotics that are commonly used against enterococcus isolates, including vancomycin (VAN), ampicillin (AMP), and linezolid (LZD). Of note, many of the enterococcus isolates (both *E. faecium* and *E. faecalis*) were resistant to vancomycin (breakpoint of >32 µg/mL), while the *E. faecium* isolates were considered intrinsically resistant to ampicillin (breakpoint ≥16 µg/mL) (Table 1). The three antibiotics were used in combination with the five different phages that exhibited lytic activity against the enterococcus isolates included in this study.

**Fig 2:**
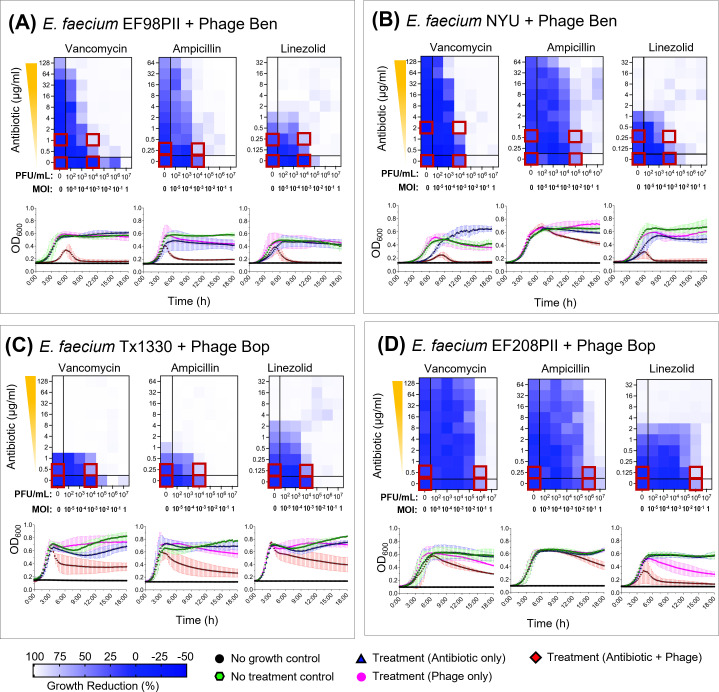
Synograms and growth curves showing the effect of various treatments (antibiotics, phages, antibiotic + phages) on growth dynamics of *Enterococcus faecium*. The color gradient in synograms represents the percentage of growth reduction. Growth reduction was calculated using following the formula: Percentage reduction = [(OD growth control – OD treatment)/OD growth control] * 100. The average of three biological replicates is shown. Antibiotics vancomycin, ampicillin, and linezolid were tested in all strains. (**A**) *E. faecium* EF98PII in the presence of phage Ben. (**B**) *E. faecium* NYU in the presence of phage Ben. (**C**) *E. faecium* Tx13301 in the presence of phage Bop. (**D**) *E. faecium* EF208PII in the presence of phage Bop. Conditions from wells marked with red squares were selected for RNAseq experiments.

**Fig 3:**
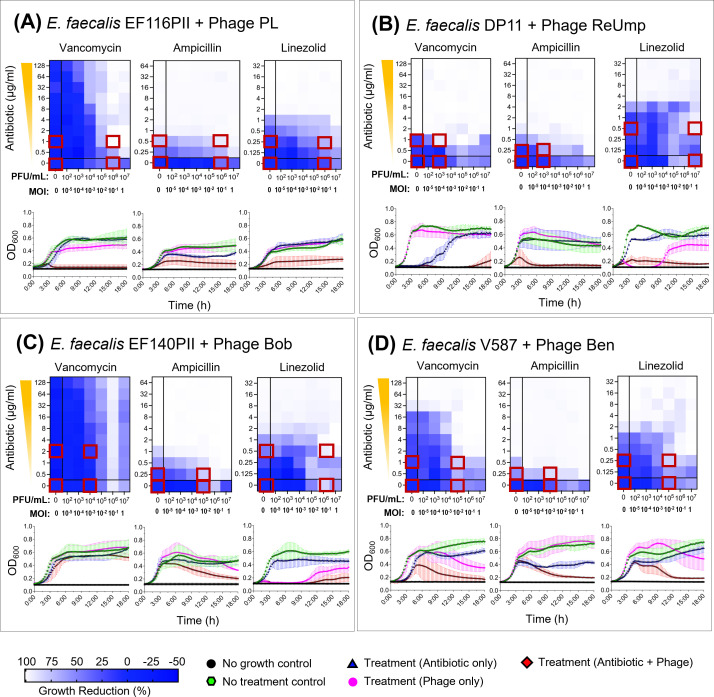
Synograms and growth curves showing the effect of various treatments (antibiotics, phages, antibiotic + phages) on growth dynamics of *Enterococcus faecalis*. The color gradient in synograms represents the percentage of growth reduction. Growth reduction was calculated using following the formula: Percentage reduction = [(OD growth control – OD treatment)/OD growth control] * 100. The average of three biological replicates is shown. Antibiotics vancomycin, ampicillin, and linezolid were tested in all strains. (**A**) *E. faecalis* EF116PII in the presence of phage PL. (**B**) *E. faecalis* DP11 in the presence of phage ReUmp. (**C**) *E. faecalis* EF140PII in the presence of phage Bob. (**D**) *E. faecalis* V587 in the presence of phage Ben. Conditions from wells marked with red squares were selected for RNAseq experiments.

In each assay, a logarithmically growing *Enterococcus* culture was incubated with a series of different concentrations of phages and antibiotics. Bacterial growth was monitored for 18 hours by measuring the OD_600_ (Fig. 2 and 3). The results were represented by phage antibiotic synergy (PAS) diagrams, which have previously been termed synograms by Liu et al. (45), where the percentage bacterial growth reduction is represented by a color gradient. We developed PAS diagrams for *E. faecium* clinical isolates EF98PII and NYU for each of the aforementioned antibiotics and phage Ben. As previously mentioned, both *E. faecium* isolates are intrinsically resistant to ampicillin (MIC ≥16 µg/mL) (Table S6) but also express high-level resistance to vancomycin (VRE) (MIC ≥32 µg/mL). When co-cultivated with phage Ben, both isolates demonstrated a growth reduction between 80% and 90% at relatively minimal levels of the antibiotics (1–2 µg/mL of vancomycin and 0.25–0.5 µg/mL of ampicillin) (Fig. 2A and B). Similar results were observed for linezolid with phage Ben, where significant reductions were observed in the antibiotic MICs when phage Ben was added (Fig. 2A and B). We observed a moderate growth reduction when laboratory-adapted *E. faecium* isolate Tx1330 was used with phage Bop for each of the antibiotics used (Fig. 2C). Minimal growth reduction was observed for the VRE clinical isolate EF208PII, phage Bop, and each of the antibiotics tested (Fig. 2D). The optimal phage titer that exhibited PAS in co-cultivation experiments was in the range of 10^4^–10^6^ PFU/mL for all the experiments. The growth reduction was significantly lower (one-way ANOVA, *P* < 0.001) in the PAS wells than in wells with only antibiotics or phages (Fig. S3; Table S1). Our data show significant growth reductions for both VRE and VSE isolates of *E. faecium* when phages are combined with antibiotics regardless of whether the enterococcus isolate had prior resistance to the antibiotic in question. High resistance levels to vancomycin did not affect whether we observed synergistic interactions between phages and vancomycin for these isolates. In addition to calculating growth reduction, Fractional inhibitory concentration (45) was also determined (Table S7). *E. faecium* EF98PII, *E. faecium* NYU, *E. faecium* Tx1330, and *E. faecium* EF208PII showed synergistic or indifferent FIC values between phages and vancomycin. *E. faecium* isolates EF98PII, Tx1330, and EF209PII showed synergistic or indifferent FIC values between phages and ampicillin; *E. faecium* NYU showed indifferent FIC values. When phages were used in combination with linezolid, synergistic FIC values were observed at low antibiotic concentrations (0.125 µg/mL for EF98PII; 0.25 µg/mL for NYU; and 1 µg/mL and below for Tx1330), antagonistic FIC values were observed at intermediate antibiotic concentrations (8 µg/mL for EF98PII, 8 µg/mL, and 32 µg/mL for Tx1330) and indifferent FIC values were observed for the rest of the concentrations.

While *E. faecium* is the most commonly observed VRE isolate in clinical medicine (46), *E. faecalis* often is capable of acquiring the same mobile genetic elements and becoming VRE. Because of the significant genetic differences between *E. faecium* and *E. faecalis* isolates, we next tested whether we observed similar trends in PAS for *E. faecalis* that we observed for *E. faecium*. We examined two separate clinical isolates EF116PII and EF140PII, along with two laboratory-adapted isolates DP11 and V587. Isolates EF116PII, EF140PII, and V587 were resistant to each of the antibiotics tested (vancomycin, ampicillin, and linezolid), while DP11 was susceptible to them all. We first tested VRE isolate EF116PII with phage PL and found that it was almost completely inhibited when only very low concentrations of vancomycin (1 µg/mL) were added (Fig. 3A). There also were moderate improvements in the inhibition of this isolate in the presence of relatively low concentrations of linezolid. All of these results were statistically significant (Fig. S3F). In this case, no inhibition improvement was observed with ampicillin. We also identified significant results for *E. faecalis* isolate DP11 in the presence of phage ReUmp and vancomycin, ampicillin, and linezolid (Fig. 3B), EF140PII in the presence of phage Bob and linezolid (Fig. 3C), and V587 in the presence of phage Ben and vancomycin and ampicillin (Fig. 3D). All of these results were statistically significant, indicating that the synergistic interactions demonstrated between antibiotics and phages were robust (Fig. S3E through H). These results indicated that many of the results observed with *E. faecium* were reproducible for *E. faecalis* and suggested a trend with enterococcus where synergistic interactions may be observed between antibiotics and phages regardless of whether pre-existing susceptibility to antibiotics was present. FIC values were not calculated for *E. faecalis* since the MIC for the phages was higher than the higher phage concentration tested in the PAS experiments.

### Transcriptomes from *E. faecium* and *E. faecalis* in PAS conditions

We sought to characterize the transcriptomes of the *E. faecium* and *E. faecalis* isolates to discern whether there were differences in gene expression profiles that might account for the synergistic responses to the phages and antibiotics. We compared the gene expression profiles in response to phages and antibiotics with those of antibiotics alone, phages alone, and with growth controls. Each of the enterococcus isolates was co-cultivated with specific phages, one of three different antibiotics, including vancomycin, linezolid, and ampicillin, or no treatment.

Because we needed relatively large quantities of RNA for transcriptome sequencing, we reproduced the experiments for *E. faecium* and *E. faecalis* at larger volumes on the conditions that were already identified as producing synergy. Three biological replicates for each of the enterococcus isolates and all phage and antibiotic combinations were obtained. Transcriptomes were obtained at 5 and 18 hours post-treatment (Fig. S4A), and the number of reads ranged from 2.5 × 10^7^ to 1.0 × 10^8^. There were generally fewer reads recovered at 18 hours compared to 5 hours, which may correspond to fewer recovered cells at the later time point (Fig. S4B).

We next clustered specimens together based on their transcriptome profiles to decipher whether patterns emerged based on antibiotics used, phages used, or a combination of both. Specimens were clustered together using principal component analysis (PCA) and were labeled according to time, antibiotic, phage treatment, and no treatment (Fig. S5). There were clear differences in expression profiles in both *E. faecalis* and *E. faecium* isolates when comparing gene expression at 5 hours and 18 hours, but there was no obvious segregation of antibiotic and antibiotic/phage groups as shown by overlapping ellipses.

**Fig 4:**
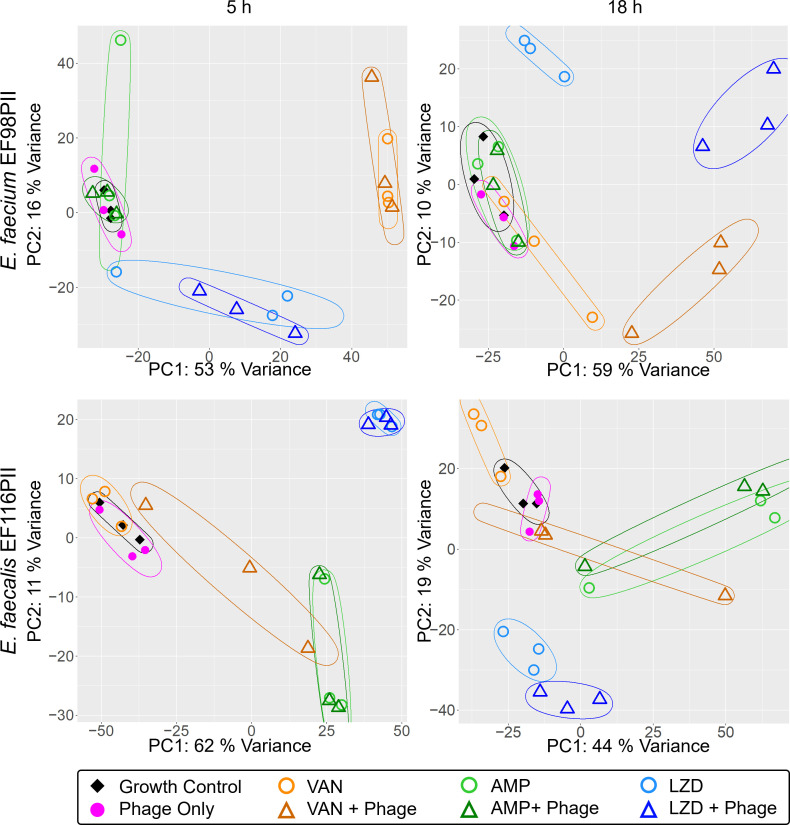
PCA shows clustering of samples at different time points and pathway analysis of PCA rotation. (**A**) Scatter plots of the first two principal components of the normalized gene expression profile of all the samples. The ellipse encircles the three biological replicates for individual experimental conditions and was drawn at a tolerance cutoff of 0.01.

Next, we segregated the isolates based on sampling time and performed PCA analysis by treatment groups to discern whether we could identify differences in gene expression profiles in phage, antibiotic, and dual antibiotic/phage synergy treatment groups (Fig. 4). For *E. faecium* EF98PII, there was significant variation among the different treatment groups at both 5 and 18 hours with PC1 of 53% and 59%, respectively; similar results were identified for *E. faecalis* EF116PII. There was distinct clustering identified when comparing the antibiotic groups to the antibiotic/phage groups at 18 hours, but not at 5 hours for both the *E. faecium* and *E. faecalis* isolates (Fig. 4). This was true regardless of the antibiotic used, but the largest segregation was observed for vancomycin and linezolid, with generally less segregation observed for ampicillin. Phage-only and growth control groups were found to be colocalized on the plot irrespective of sampling time.

### Phage-antibiotic synergy alters cellular stress responses and membrane transport

**Fig 5:**
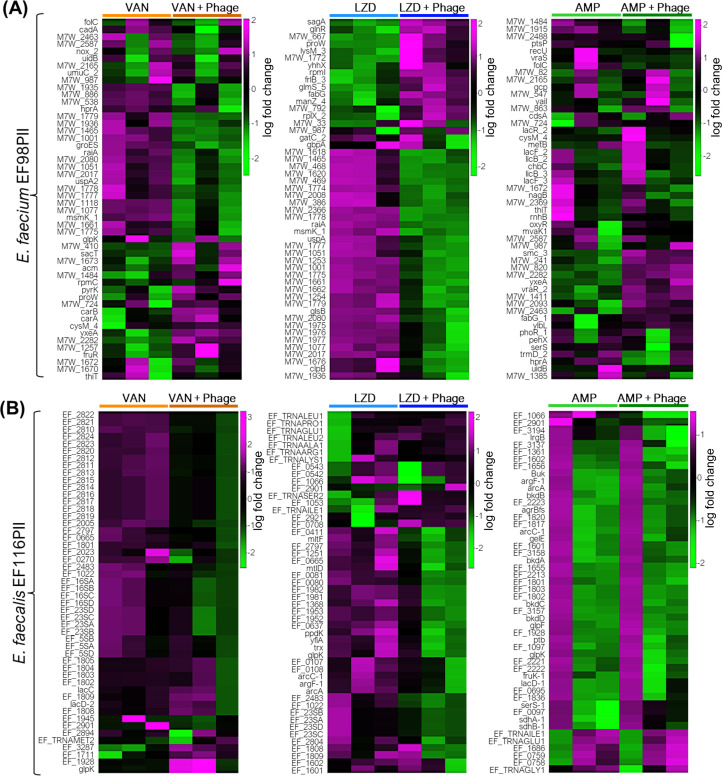
Heatmap shows the top 50 differentially expressed genes. in *E. faecium* EF98PII (**A**) and *E. faecalis* EF116PII (**B**) for various comparisons between “Antibiotic +Phage” versus “Antibiotic” only treatment samples at 18 hours post co-culture. The data were centered by subtracting the average expression level for each gene and samples were normalized by dividing with SD. Pearson’s correlation coefficient and average linkage were used to calculate the distance matrix. Each sample group includes three biological replicates and is highlighted by colored bars atop the heatmaps. The color key legend next to each heatmap represents the degree of variation in the expression of genes between samples.

Because there was clear delineation in gene expression profiles when comparing antibiotics versus the combined effects of antibiotics and phages at 18 hours compared to 5 hours, we focused our analysis on the 18 hour time point moving forward to identify differences that might account for the synergistic effects observed. We performed differential gene expression analysis using iDEP v0.96 to identify the top 50 differentially expressed genes between the antibiotic only and the antibiotic/phage synergy. We found that the majority of top differentially expressed genes were involved in membrane transport, DNA replication and damage repair, transcription regulation, and cellular stress response regulation (Fig. 5; Tables S2 and S3). There is a distinct separation between the over-expressed and under-expressed genes in the vancomycin and linezolid treatment groups, while the distinction is less in the ampicillin treatment group (Fig. 5). While ampicillin and vancomycin are cell wall inhibitors, their binding targets are different (47, 48). This may have an effect on the response to PAS observed between the two antibiotics

In the presence of vancomycin and phages in *E. faecium* isolate EF98PII, some of the highly expressed genes encode proteins involved in membrane transport systems. Some of those genes include the following: Cadmium-translocating P-type ATPase (*cadA*), manganese transport protein (mntH), ABC transporter ATP-binding protein (*msmK_1*), substrate-binding domain of ABC-type glycine betaine transport system (*proW*), PTS system (fructose-specific IIa, IIB, IIC components), hydroxymethylpyrimidine ABC transporter (transmembrane component), and energy-coupled thiamine transporter ThiT (*thiT*). Similarly, heat shock protein Hsp20, universal stress protein UspA2, and co-chaperone (*groES*) involved in regulating stress responses and protein folding were among the top 50 differentially expressed genes (Table S2). In addition, genes involved in membrane transport systems, stress response, and ribonucleoprotein and protein biosynthesis were found to be expressed under both ampicillin/phage and linezolid/phage treatment conditions in *E. faecium* EP98PII (Table S2).

For *E. faecalis* isolate EF116PII, we identified many of the identical sets of highly differentially expressed genes that were also identified in *E. faecium* isolate EF98PII (Tables S2 and 3), indicating that despite the significant genetic differences between the two species, the responses to the antibiotics and phages were highly similar. We also identified additional differentially expressed genes in *E. faecalis* EF116PII. Some of these were related to prophages, as they represented a major capsid protein (EF_2820), minor structural protein (EF_2811), tail tape measure protein (EF_2813), and major tail protein (EF_2815), and were all downregulated in response to the combination of vancomycin/phage (Fig. 5B; Table S3).

We next used DeSeq2 to characterize differentially expressed genes between the antibiotic and the phage/antibiotic conditions to determine whether similar results were identified by different methods and whether additional differentially expressed genes could be identified. We used a minimum fold-change value of 2 with a minimum false discovery rate (FDR) of 0.05. We found that a total of 352/329 and 304/210 genes were significantly (*P*adj <0.05) upregulated/downregulated in *E. faecium* EF98PII and *E. faecalis* EF116PII, respectively, when the vancomycin/phage treatment group was compared to the vancomycin group. Similarly, 464/465 and 10/34 genes were found to be significantly (*P*adj <0.05) upregulated/downregulated in *E. faecium* EF98PII and *E. faecalis* EF116PII, respectively, in the linezolid/phage treatment compared to the linezolid group (Fig. 5A). However, as suggested by the low degree of variability between ampicillin/phage and ampicillin groups (Fig. 4), we see a relatively small pool of genes significantly (*P*adj <0.05) upregulated/downregulated (11/1) in *E. faecium* EF98PII and none in the case of *E. faecalis* EF116PII (Fig. 5A).

There were a number of different genes identified in *E. faecium* EF98PII using DeSeq2 that were highly upregulated under vancomycin/phage synergistic conditions compared to vancomycin alone (Table S4). These include a transcriptional repressor of the fructose operon (M7W_1255, fruR), a transcription anti-terminator (M7W_2638, *sacT*), an oligopeptide transport system permease protein (M7W_2290; *oppB*), a PTS system, fructose-specific component (M7W_1257), and sortase A (M7W_698) among others (Table S4). There also were a number of significantly downregulated genes in vancomycin/phage synergistic conditions compared to vancomycin alone. These included a number of hypothetical genes, organic hydroperoxide resistance (M7W_1936), heat shock protein Hsp20 (M7W_2017), multiple sugar ABC transporter, manganese transport protein MntH (M7W_1001), universal stress protein family (M7W_1568, *uspA2*), ATP-binding protein (M7W_2275, *msmK_1*), cadmium-translocating P-type ATPase (M7W_1465), and others (Table S4).

We also examined those genes that were differentially regulated in *E. faecium* EF98PII at 18 hours using DeSeq2, focusing on those that were significantly upregulated under linezolid/phage synergistic conditions compared to linezolid alone. These genes included a D-serine, D-alanine, glycine transporter (M7W_814), sortase A (M7W_73), oligopeptide transport system permease (M7W_2290, *oppB*), ABC transporter membrane-spanning permease glutamine transport (M7W_2302, *yecS*), and a glycine betaine ABC transport permease (M7W_2392, *proW*), among others (Table S4). We also identified some downregulated genes, which included heat shock protein Hsp20 (M7W_2017), an N-acetylglucosamine-specific IIA, IIB, IIC component (M7W_1488), a cadmium-translocating P-type ATPase (M7W_1465), a manganese transport protein MntH (M7W_1001), a universal stress protein family (M7W_1773, *uspA*), a multiple sugar ABC transporter, an ATP-binding protein (M7W_2275, *msmK_1*), an abortive infection protein (M7W_2008), a universal stress protein family (M7W_469), and a putative hydrolase of alpha, beta superfamily (M7W_1662), among others (Table S4).

When examining the effects on *E. faecium* EF98PII of ampicillin/phage synergy compared to ampicillin alone at 18 hours, we identified fewer genes that had altered regulation compared to linezolid and vancomycin (Table S4). We identified upregulation in a transporter-associated gene associated with vraSR (M7W_1411) and a response regulator (M7W_1413, *vraR_2*). We identified downregulation in other hypothetical genes (Table S4).

We also characterized *E. faecalis* EF116PII using DeSeq2 to identify overlapping upregulated or downregulated genes between *E. faecalis* (EF116PI) and *E. faecium* (EF98PII) during phage and antibiotic selection. We began by examining those genes that were significantly upregulated under vancomycin/phage synergistic conditions compared to vancomycin alone (Table S5). Those genes included an ABC transporter ATP-binding protein (EF_1333), a site-specific integrase (EF_0479), and an amino acid permease (EF_1103). Downregulated genes included a phage-associated holin (EF_2803/2804), a phage tail protein (EF_2005), a phage baseplate upper protein (EF_2810), an HK97 gp10 family phage protein (EF_2007), a PTS transporter subunit EIIC (EF_0270), and a PTS sugar transporter subunit IIA (EF_0412, *mltF*), among others.

We next examined the gene expression responses of *E. faecalis* EF116PII to linezolid/phage synergy compared to linezolid alone (Table S5) using DeSeq2 to identify upregulated and downregulated genes. We identified many upregulated genes in response to linezolid/phage synergy that mostly represented transcriptional regulators and ABC transporters. These genes included ABC transporter ATP-binding protein (EF_2652, *potA*), ABC transporter substrate-binding protein (EF_2649), ABC transporter permease (EF_2650/2651), ABC transporter ATP-binding protein (EF_1673), Sigma-54-dependent transcriptional regulator (EF_1010), and response regulator transcription factor (EF_0926), among others (Table S5). Other genes were downregulated in response to linezolid/phage synergy, which included mostly membrane transporters, phage-associated genes, toxin-antitoxin systems, and sugar transporters. These genes included PTS sugar transporter subunit IIA (EF_0412, *mltF*); PTS mannitol transporter subunit IICBA (EF_0411), phage-associated protein holin (EF_2803), phage tail protein (EF_2001/2005), phage baseplate upper protein (EF_2810), phage tape measure protein (EF_2003), type II toxin-antitoxin system RelB/DinJ family (EF_0512), and type II toxin-antitoxin system YafQ family (EF_0513).

### Pathway analysis

**Fig 6:**
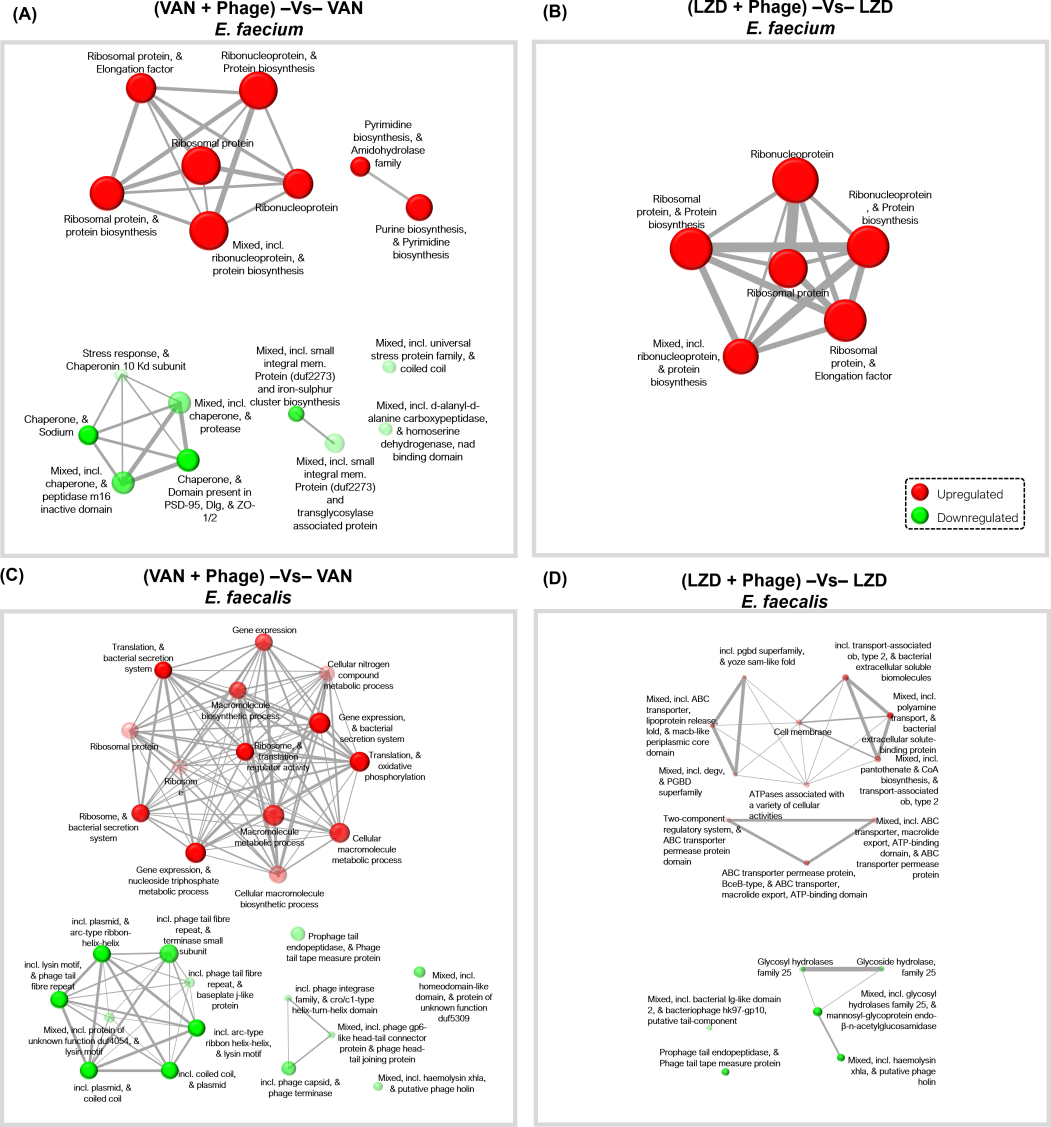
Pathway analysis of significantly upregulated/downregulated genes during “Phage + Antibiotic” co-culture as compared to “Antibiotic” only culture at 18 hours. Network of pathways involving differentially expressed genes isolated from *E. faecium* EF98PII (**A, B**) and *E. faecalis* EF116PII (**C, D**) treated with “Antibiotic + Phage” versus “Antibiotics” only at 18 hours. Genes were filtered as background and “all available gene sets” were used for enrichment analysis of differentially expressed genes. Two pathways (nodes) are connected if they share 30% or more genes. Red represents upregulated and green represents downregulated. The node sizes correspond to their respective gene set sizes. Darker-colored nodes correlate to more significantly enriched gene sets. Thicker edges represent more overlapped genes.

We next used the STRING database (49) to identify biosynthetic pathways that may be involved in antibiotic/phage synergy responses. We first evaluated the response in *E. faecium* EF98PII and found that pathways involved in purine and pyrimidine biosynthesis were significantly enriched in both the vancomycin/phage (Fig. 6A) and linezolid/phage (Fig. 6B) responses compared to antibiotics alone. By contrast, we identified various stress response pathways that were inhibited, along with suppression of chaperones, membrane transport channels, and iron-sulfur cluster biosynthesis. In response to ampicillin/phage synergy, fewer pathways were identified. These included a small number of upregulated genes involved in cell-wall biosynthesis, phage shock protein C, and transcription regulators such as Helix-turn-helix domain *rpiR*.

We also used the STRING database to identify biosynthetic pathways that may be involved in the responses to antibiotic/phage synergy in *E. faecalis* EF116PII. Most of the gene pathways being downregulated when vancomycin/phage was present were associated with phages, while biosynthetic pathways were significantly enhanced (Fig. 6C). In response to linezolid/phage synergy PGBD-like superfamily gene pathways involved in peptidoglycan binding, and ABC transporter permease pathways that confer antibiotic resistance in gram-positive bacteria were upregulated (Fig. 6D).

## DISCUSSION

There is a significant need to identify alternatives for the treatment of enterococcal infections in humans. The organism has the potential to resist many commonly used antibiotics through the acquisition of a plasmid (such as vanA or vanB), a transposon, or through the presence of genes on the genome (such as vanD) in certain species. Although vancomycin has historically been a critical component of most enterococcal treatment regimens, an increasing prevalence of VRE over the past decade has mandated the development of alternative treatment approaches. Patients who have received extensive antibiotic therapy including bone marrow and organ transplant recipients, are chronically ill, or are in long-term hospital care facilities, are at particular risk from VRE infections. In these patients, the enterococci can be extremely difficult to eradicate and often recur following cessation of therapy. Thus, alternative treatments, such as phage therapy, and protocols for decolonization of these patients of their enterococcus infections have become increasingly attractive options.

Phages have become alternative and adjunctive therapies to antibiotics for multidrug-resistant bacteria but have been difficult to deliver in a timely fashion to patients in acute clinical situations. Part of this has been due to the manner in which phages are evaluated to determine whether they may be efficacious against the pathogens that are associated with disease severity. Generally, pathogens such as enterococci are identified as causing an illness, and then those pathogens are referred to centers that are willing to screen for phages that are capable of lysing those pathogens. Generally, those centers will identify whether those phages are capable of killing the pathogens with the efficiency of plating values (50). This procedure generally ignores the fact that the phage and or phages that will be delivered to the patient will almost never be delivered alone without the concomitant delivery of standard-of-care antibiotics. Thus, we sought to characterize a more realistic situation that more accurately reflects the situation that is observed clinically when patients receive phage therapy. That is, what is observed when the patient receives both phages and antibiotics simultaneously.

We evaluated the impact of phages and antibiotics when used concurrently extended and confirmed prior observations. Those findings were that antibiotics such as vancomycin could work synergistically with phages to eliminate the enterococci (46, 51). Our results extended those findings by demonstrating that this was true for both *E. faecium* and *E. faecalis*, for clinical isolates and laboratory-adapted isolates of enterococci, and most importantly, that synergy was observed whether or not the enterococcal isolate was antibiotic susceptible. For example, we observed significant synergistic interactions with ampicillin and phages in *E. faecium*, which is a combination that would almost never be used clinically because *E. faecium* is considered to be intrinsically resistant to ampicillin (52). Perhaps more importantly, we confirmed in our clinical isolates of VRE *E. faecium* and *E. faecalis* that despite their confirmed high-level resistance to vancomycin (Table 1) when combined with phages, the MICs to vancomycin were reduced to within readily achievable ranges (Fig. 3). This has been observed in other studies for *E. faecium* (40, 53). Such a finding has significant implications for the clinical treatment of recalcitrant VRE infections as well as decolonization protocols for VRE, as it indicates that antibiotics that had previously been deemed useless against this MDR pathogen may have significant utility when used in combination with phages.

It is important to note that the antibiotic resistance in this study that was overcome through the process of synergy was not a permanent phenomenon. Indeed, we tested some of these isolates afterward just to demonstrate that they were still antibiotic resistant (Table S6) despite the fact that they had susceptible MICs when they were tested in the presence of both phages and antibiotics. This was important because it indicated that the combined effect of the antibiotic and the phage required the ongoing presence of phages. Upon removal of the phages, the high-level ampicillin or vancomycin resistance returned. This is contrary to a situation that has been observed with other phages with organisms such as *Pseudomonas aeruginosa* (54, 55) and *Acinetobacter baumanii* (56) where the utilization of certain phages leads to more permanent changes in antimicrobial susceptibilities that are not reversed with the removal of the phages.

We expected to find combined effects of phages and antibiotics when we treat enterococci with both simultaneously. Indeed, we identified these phenomena when we developed a solid media assay for assessing the combined effects of antibiotics and phages on multiple different bacteria, including *E. faecium* and *E. faecalis* (57). What is surprising to us is the ability to overcome these phenomena in clinical VRE isolates that are confirmed to have *vanA*-expressing plasmids. These plasmids are known to express high levels of the *vanA* genes that result in the proteins saturating the cell walls of the enterococcal cell surfaces, resulting in high levels of resistance to vancomycin, which no longer bind to these altered VanA (58). We did not expect to restore vancomycin susceptibility in these organisms through the addition of a phage, as one might assume the restoration might occur through the downregulation of *vanA* (which was not observed), or the upregulation of the native gene (which also was not observed). Thus, a complex combination of regulatory factors involving nucleotide and protein biosynthesis, stress responses, and transport appear to be involved in the synergy responses rather than just a reversal of the original antibiotic resistance response. While we could not pinpoint the exact mechanism responsible for the restored susceptibility to the antibiotic and phage using transcriptomic analysis, a study such as this one is just the first step in elucidating what may be necessary to pinpoint the mechanism behind developing synergistic responses between antibiotics and phages.

## MATERIALS AND METHODS

### Bacterial strains, growth conditions, and quantification

*Enterococcus* isolates used in this study were collected from patients admitted at the University of California San Diego (UCSD) Health. Four different strains each of *E. faecium* and *E. faecalis* were used to study the synergistic interaction between antibiotics-phages and their antibiotic resistance profiles were determined using broth microdilution techniques using BD Phoenix instrument (Becton Dickinson, Franklin Lakes, NJ, USA) using standard susceptibility cutoffs (59) (Table S6) at the UCSD Centre for Advanced Laboratory Medicine (Table 1). For each species, we tested three VAN-resistant and one VAN-sensitive strain against a combination of their respective phages with antibiotics: (vancomycin [VAN], ampicillin [AMP], and linezolid [LZD]) or antibiotics only to screen for antibiotic-phage synergy treatment condition (Table 1). All bacterial strains were cultured in Brain Heart Infusion (BHI) medium (BD Difco, Catalog# DF0418-17-7) at 37°C with shaking at 200 rpm (for liquid culture) and supplemented with antibiotics during synergy experiments. For bacterial propagation and maintenance, solid BHI media was used with 1.5% agar while for plaque assays 0.4% top agar was used over the solid media. For quantification, 100 µL of diluted bacterial samples from each treatment condition (usually dilution 10^−4^, 10^−5^, and 10^−6^) was spread onto 1.5% BHI agar plates and incubated overnight at 37°C. The following day, the number of colonies was counted on each dilution plate and the colony forming units per ml (CFU/mL) for each sample was determined.

### Bacteriophage isolation, propagation, quantification, and characterization

The phages used in this study (Table 2) were isolated from sewage samples from different regions of southern California using multiple enrichment protocols as described elsewhere with some modifications (60). Briefly, sewage samples were centrifuged at 10,000 × *g* for 10 minutes at 4°C to remove particulate matter and then 20 mL supernatant was mixed with an equal volume of double strength (2×) BHI broth. To this mixture, 500 µL of overnight grown culture of *E. faecium* or *E. faecalis* (diluted to OD_600_ ~0.2) was added and incubated overnight at 37°C in a shaker incubator. One percent vol/vol chloroform was added to the mixture, vortexed, and incubated at room temperature (RT) for 30 minutes. This was followed by centrifugation at 5,000 × *g* for 15 minutes at 4°C and supernatant was filtered through sterile 0.45 µm PVDF syringe filters (Whatman Puradisc, Item# 6746–2504). Next, to screen for phages against these bacterial species, spot assays were performed as follows. An amount of 100 μL of overnight grown culture of enterococcus (diluted to OD_600_ ~0.2) was mixed with molten BHI top agar (cooled to ~45°C) and uniformly spread over the BHI agar plates. After the soft agar was solidified, 5 µL of filtered phage lysate (from different sewage samples) was spotted on their respective bacterial species plates. Spots were air-dried followed by overnight incubation of plates at 37°C. Plates were examined for clear spots and if positive, they were further processed for three rounds of phage purification by plaque assay as described by Wandro et al. (35). Briefly, clear spots were picked by 1 mL sterile pipette tip and resuspended in 100 µL sterile PBS buffer. Following brief vortexing and centrifugation, the supernatant was mixed with an equal volume of *Enterococcus sp*. culture (OD_600_ ~0.2) and incubated at 37°C for 10 minutes. Molten BHI top agar (5 mL; cooled to ~45°C) was added to this mixture and immediately spread over the BHI agar plate. Following overnight incubation at 37°C, plaques were further purified by repeating the plaque assay step (as mentioned above) two more times. Phage stock solutions were prepared by growing purified phages with their respective enterococcus hosts in 25 mL BHI broth in a shaker incubator (200 RPM) maintained at 37°C overnight. Cultures were vortexed and then centrifuged at 10,000 × *g* for 10 minutes followed by supernatant filtration using a 0.2 µm syringe filter. For long-term storage, phages were stored at −80°C in a BHI medium with a 25% glycerol solution. To determine the phage titer, a plaque assay was performed with their respective experimental strains. Phage stocks were serially diluted up to 10^−8^ dilution using BHI broth and 5 µL from each dilution was spotted on BHI agar plates with an overlay of top agar containing host bacteria. Based on the plaque assay results, the titer of each phage (PFU/mL) was determined. Phage titers as well as efficiency of plating (EOP) values in testing strains are shown in Table S8. Three of the total five phages used in this study (Table 2) were characterized previously by Wandro et al. but the phage PL and ReUmp were isolated, purified, and characterized during this study.

### Phage and bacterial genome sequencing and analysis

Total genomic DNA from phages PL and ReUmp and bacterial isolates were extracted using QIAamp UltraSens Virus kit (Qiagen catalog# 53706) and DNeasy Blood & Tissue Kit (Qiagen catalog# 69504), respectively. The quality of the extracted DNA was checked using a Qubit dsDNA high-sensitivity assay kit (Invitrogen, catalog# Q32851), and DNA libraries were prepared using a Nextera XT DNA library preparation kit (Illumina, catalog# FC-131–1024). Paired-end sequencing (2 × 150 bp) was used to sequence the whole genome of the phage PL on the Illumina iSeq100 platform and the bacterial genomes were sequenced on the Illumina Miseq platform. Sequencing reads were assembled into scaffolds using the DeNovo approach of CLC Genomics Workbench software version 21.0.3 (Qiagen, Redwood City, CA, USA). Next, genomes were annotated using an online open-source annotation tool RASTtk v2.0 (Rapid Annotation using Subsystem Technology tool kit (61). A complete genome of the phage was visualized using PROKSEE analysis using CGView Server (62).

### Transmission electron microscopy

TEM staining and analysis were performed as previously described by Lee et. al.*,* with some modifications (63). Briefly, 10 µL drops of concentrated phage solution (~2 × 10^8^ PFU/mL) was spotted on a clean parafilm sheet. Carbon-coated copper grids (PELCO SynapTek Grids, product# 01,754 F) were placed over the drops for approximately 1 minute followed by three passes over the 20 µL drops of sterile deionized water and excess water was blotted using filter paper. Finally, the grids were negatively stained by placing over 10 µL drop of 2% uranyl acetate solution (pH 4.0) for ~45 seconds. The grids were then immediately blotted using filter papers and air-dried at room temperature for 5 minutes. Grids were imaged after at least 24 h of staining using Joel 1400 plus at the University of California, San Diego—Cellular and Molecular Medicine Electron Microscopy Core facility (RRID:SCR_022039).

### Phage-antibiotic synergy testing

PAS testing was performed in a BHI medium as previously described by Liu et. al.*,* with some modifications (42). The experiment was setup in a 96-well plate in a total volume of 200 µL growth medium containing varying concentrations of antibiotics and phages (Fig. 2). The antibiotics and phages were serially diluted 10-fold from top to bottom and from right to left for antibiotics and phages, respectively. This created a concentration gradient for antibiotics only (1st column), phages only (2nd row from bottom), and antibiotic/phage (central wells). Similarly, bacterial growth control and no growth control were also set up in triplicate wells (Fig. 2). Briefly, a single bacterial colony was inoculated in 2 mL BHI broth followed by overnight incubation in a shaker incubator maintained at 37°C and 200 rpm. The next morning, the culture was diluted in BHI broth (1:400) followed by a short incubation of ~3 h to get exponentially growing bacterial cells. The culture was then adjusted to OD_600 =_ 0.1 (~ 1 × 10^8^ CFU/mL) by diluting in BHI broth, and 20 µL of the diluted culture was added to each well of the 96-well plate containing 140 µL of BHI broth to yield a final concentration of ~1 × 10^7^ CFU/mL in each well. Next, the phage stock solution was serially diluted 10-fold in BHI broth ranging from ~1 × 10^8^ to 1 × 10^3^ PFU/mL and 20 µL from each dilution was added to each well of column B to column G, respectively. This resulted in a 10-fold reduction in phage concentration in each column compared to their respective diluted phage stock samples. To be specific, each well of column B had a final concentration of ~1 × 10^7^ PFU/mL and each well of column G had a final concentration of ~1 × 10^2^ PFU/mL. This established a range of multiplicity of infection from 1 to 10^−5^ (MOI, which is defined as the ratio of the numbers of phage particles to the numbers of the host cells) across the columns (B to G) from 1 to 1 × 10^−5^. This was followed by the addition of 20 µL of serially diluted antibiotic stock solutions to each well which resulted in a final concentration ranging from 128 to 0.5 µg/mL (VAN), 64 to 0.25 µg/mL (AMP), and 32 to 0.125 µg/mL (LZD) from rows 2 to 10 (Fig. 2). The plates were incubated in a VERSAmax Microplate Reader for 18 hours and OD_600_ was measured every 15 minutes after shaking for 3 seconds. Antibiotic stock solutions were prepared fresh in ultrapure sterile water, filter sterilized using 0.22 µm filters, and stored at 4°C. The PAS experiment was performed in three biological replicates. The PAS was identified based on the percentage growth reduction of bacteria in each well which was determined using the following formula as previously described(42).


$$Percentage\;reduction=\;100\;x\;\frac{OD_{600\;}\;growth\;control-\;OD_{600\;}treatment}{OD_{600}\;growth\;control}$$

FIC was calculated as previously described (45) for *E. faecium* experiments. FIC values were not calculated for *E. faecalis* since phage MIC was not obtained.

### Sampling scheme for RNA isolation and sequencing

To harvest bacterial samples for transcriptomic analysis, coculture assays were repeated in higher culture volume for specific growth conditions for *E. faecium* EF98PII and *E. faecalis* EF116PII which exhibited synergistic interaction during co-culture assays (see Fig. 2 and 3 red squares for synergy conditions). Plates containing 24 wells (Thermo Scientific Nunc Non-Treated Multidishes, catalog# 144530) were used for setting up co-culture assay in a total volume of 600 µL BHI broth for each synergy combination along with proper controls for 5 hour and 18 hour time points. This experiment was repeated on three separate days that represented three biological replicates for each sample. Samples (16 samples × 3 biological replicates, *n* = 48) were collected for each of the two bacterial strains which added to a total of 96 samples as shown in the sampling scheme (Fig. 4A). From each well, 450 µL sample was pooled into separate Eppendorf tubes and total RNA was extracted using the RNeasy Protect Mini Kit (Qiagen, catalog# 74124). Isolated RNA samples were quantified using both Qubit RNA BR assay kit (Invitrogen, catalog# Q10210) and NanoDrop 2000/2000c (Thermo Fisher Scientific, catalog# ND2000CLAPTOP). Approximately 880 ng of RNA was used to build a cDNA sequencing library using the Illumina stranded total RNA prep with ribo zero plus kit (catalog# 20040529). 100 bp paired-end sequencing was performed on the Illumina NovaSeq 6000 platform using S4 flow cell (Illumina Inc., San Diego, CA, USA).

### Differential gene expression analysis

RNA sequencing analysis was performed using command line tools and R-studio (v2022.12.0 + 353). Initially, demultiplexed raw sequencing reads were trimmed using Trimmomatic (v0.39) with the following parameters: ILLUMINACLIP:TruSeq3-PE.fa:2:30:10 LEADING:3 TRAILING:3 SLIDINGWINDOW:4:15 MINLEN:35 (64). Reference genome file (FASTA format) and gene annotation files (GFF format) were downloaded from the Nucleotide Sequence Database of NCBI for *Enterococcus faecium* NRRL B-2354 and EnsemblBacteria for *Enterococcus faecalis* V583. Reference genomes were indexed using HISAT2 (2.0.5) followed by sequencing read mapping (65). Next, transcripts were assembled from mapped reads and reference transcript annotation files by StringTie (66), and transcript abundance in each sample was estimated and counted to identify differentially expressed genes using a Ballgown tool (67). Finally, the DESeq2 algorithm of the free online server, iDEP.96 was used to calculate fold change values for differentially expressed genes (68). A minimum value twofold change was considered to filter out significantly upregulated and downregulated genes with an FDR cutoff value of less than 0.05. Finally, default parameters were used to perform pathway analysis using the Gene Ontology (GO) database (69) and STRING: a database of predicted functional associations between proteins (70).

### Statistical analysis

All the experiments were performed in three independent biological replicates. The data shown in the heatmaps represent the mean percentage reduction of bacterial growth that originated from three biological replicates. Growth curve figures and bar graphs show the means and standard deviations (SDs) of three biological replicates. To determine statistical significance one-way ANOVA was performed using Dunnett’s multiple comparisons test on GraphPad Prism 9 (v9.2.0) between treatment and control groups and a *P*-value of <0.05 represented that the given data are significant.

## Data Availability

Sequencing data are available in NCBI SRA under BioProject accession no. PRJNA947989 (96 samples), BioSample no. SAMN33872066 to SAMN33872161 (RNA sequences of 96 bacterial samples), BioSample no. SAMN36748519, SAMN36748517, and SAMN36748518 (whole-genome sequences of three bacterial strains), and Biosample no. SAMN36749450 (whole-genome sequences of Phage PL).

## SUPPLEMENTAL MATERIAL

Supplemental figures aac.00740-24-s0001.pdf Figures S1 to S5.

Table S1 aac.00740-24-s0002.xlsx One-way ANOVA test scores for different treatments.

Table S2 aac.00740-24-s0003.xlsx Differential expression for E. faecium EF98PII.

Table S3 aac.00740-24-s0004.xlsx Differential expression for E. faecalis EF116PII.

Table S4 aac.00740-24-s0005.xlsx Fold change of genes in E. faecium EF98PII (VAN+Phage-Vs-VAN_18h).

Table S5 aac.00740-24-s0006.xlsx Fold change of genes in E. faecalis EF116PII (VAN+Phage-Vs-VAN_18h).

Table S6 aac.00740-24-s0007.xlsx Antibiograms of strains used in this study.

Table S7 aac.00740-24-s0008.xlsx Fractional inhibitory concentration.

Table S8 aac.00740-24-s0009.xlsx Phage titer in production and testing strains.

ASM does not own the copyrights to Supplemental Material that may be linked to, or accessed through, an article. The authors have granted ASM a non-exclusive, world-wide license to publish the Supplemental Material files. Please contact the corresponding author directly for reuse.
