# Supplementary material for: In vitro resensitization of multidrug-resistant clinical isolates of Enterococcus faecium and E. faecalis through phage-antibiotic synergy
Source: Antimicrob Agents Chemother. 2024 Dec 19;69(2):e00740-24. doi: 10.1128/aac.00740-24 (PMC11823633; doi:10.1128/aac.00740-24)
Supplement: Supplemental figures — Figures S1 to S5. [file aac.00740-24-s0001.pdf]

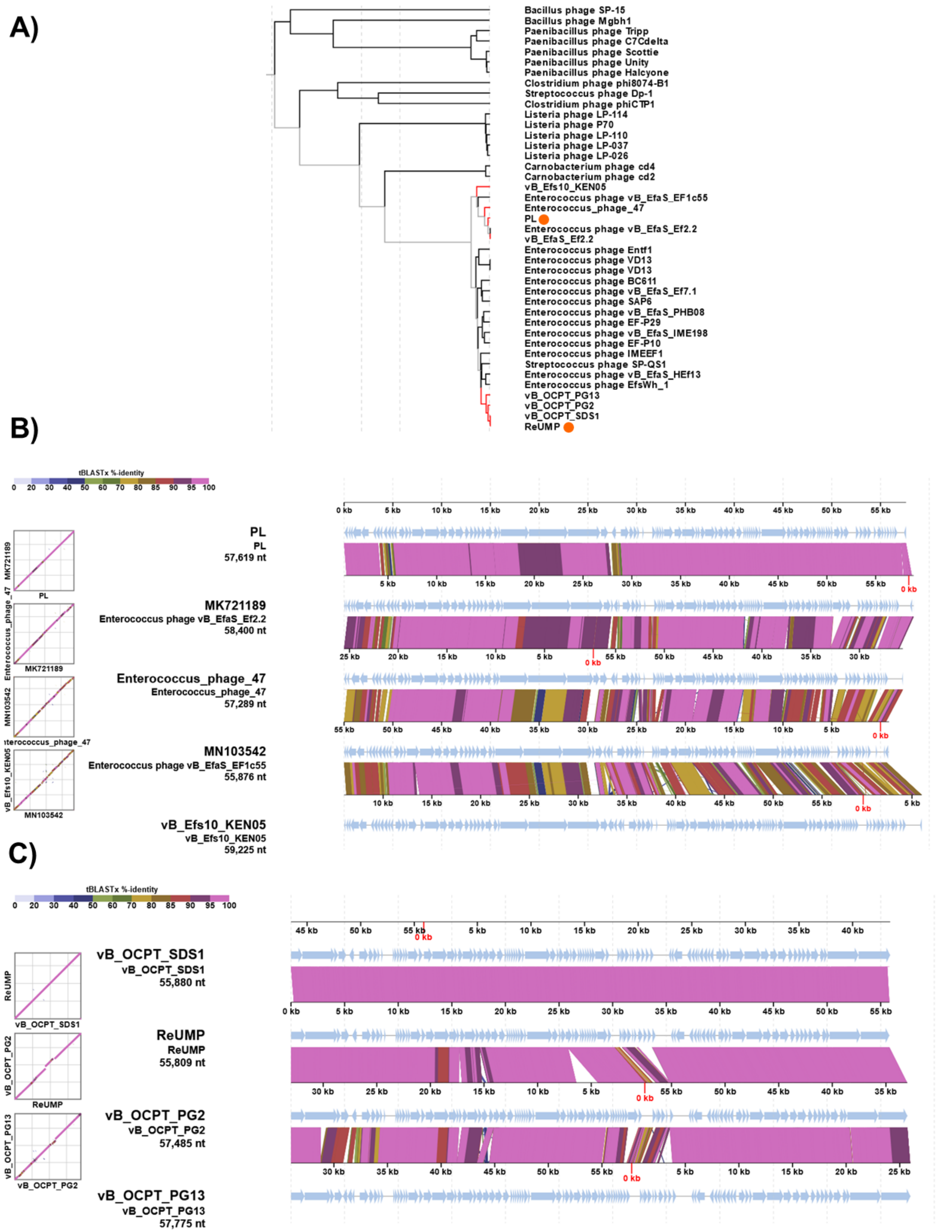

Figure S1. Genomic comparisons of phage PL and ReUmp. Phage genomes were clustered with related genomes using the Phage Proteomic Tree implemented in the VIPtree server (A). Genome maps to the closely related genomes to phage PL (B) and phage ReUmp (C).

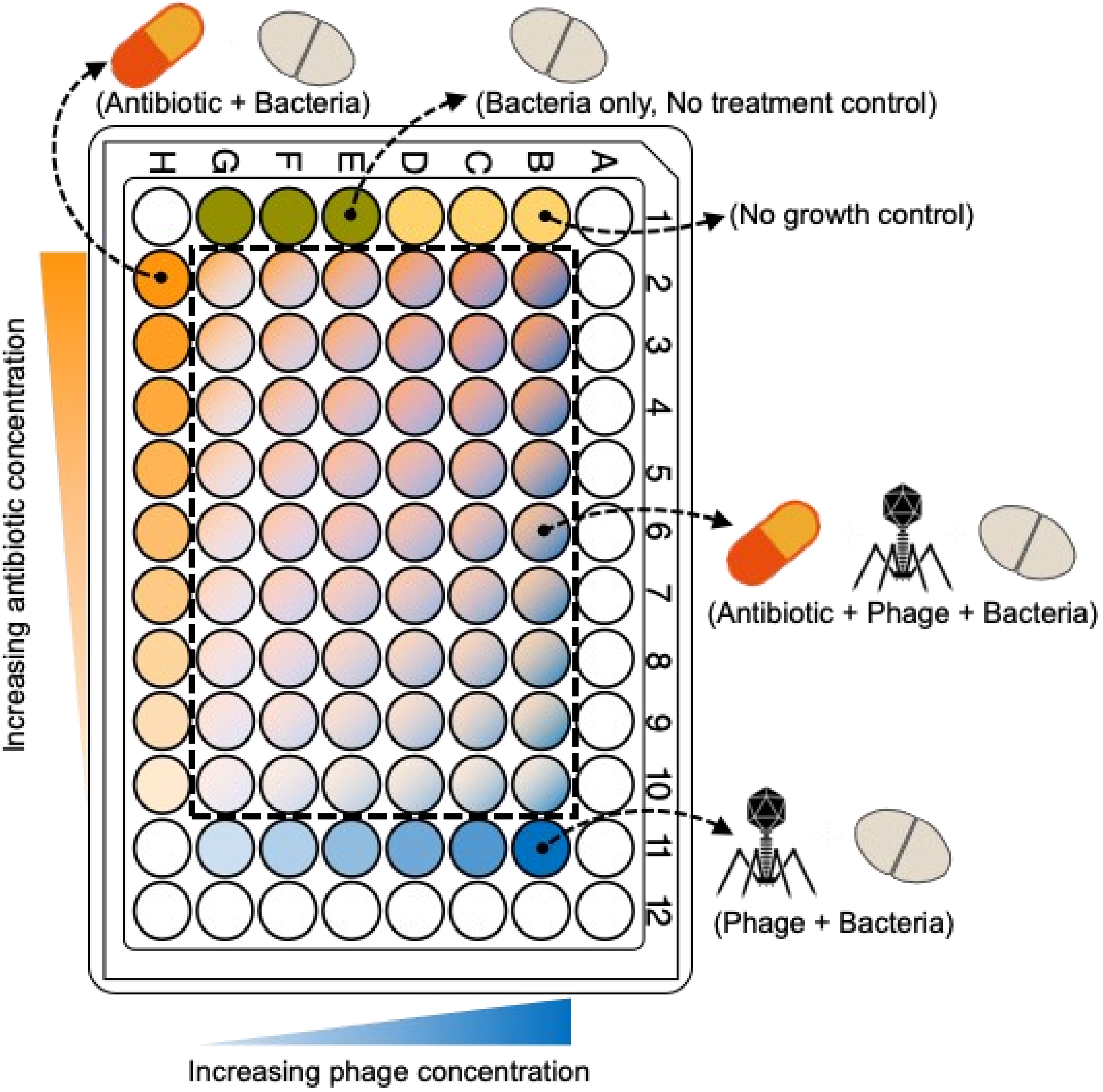

Figure S2. Experimental layout of the phage-antibiotic co-cultivation assay. Bacterial growth in the presence of antibiotics, phage, or both were studied using a 96 well plate. Differently colored wells represent various treatments: Antibiotic, phage, antibiotic + phage, no treatment control, and no growth controls. Color intensity in the individual wells represent their respective concentration gradient. Some outer wells (colorless) bordering the plate were unused and were filled with BHI media.

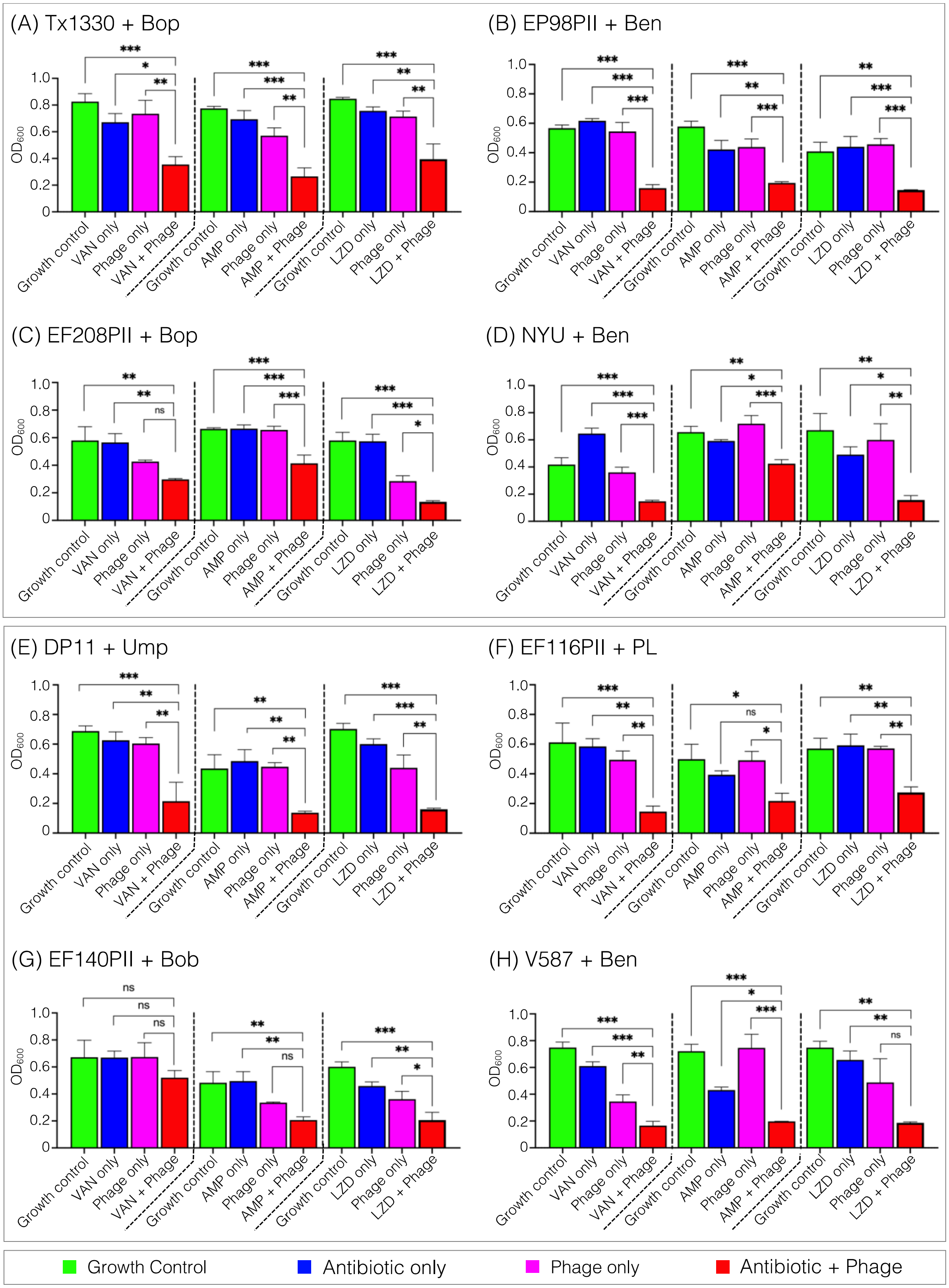

Figure S3. Bar graph shows average optical density (OD_{600}) with standard deviation (+/-) for synergistic interaction between phage and antibiotic at 18 hours. Data are shown only for the specific combinations that are highlighted in red boxes on heatmaps in Figure.3 against various *E. faecium* strains. One way ANOVA test was performed to determine the percentage of significance (*** P - value < 0.0001, ** P - value < 0.001, * P - value < 0.01, ns - not significant)

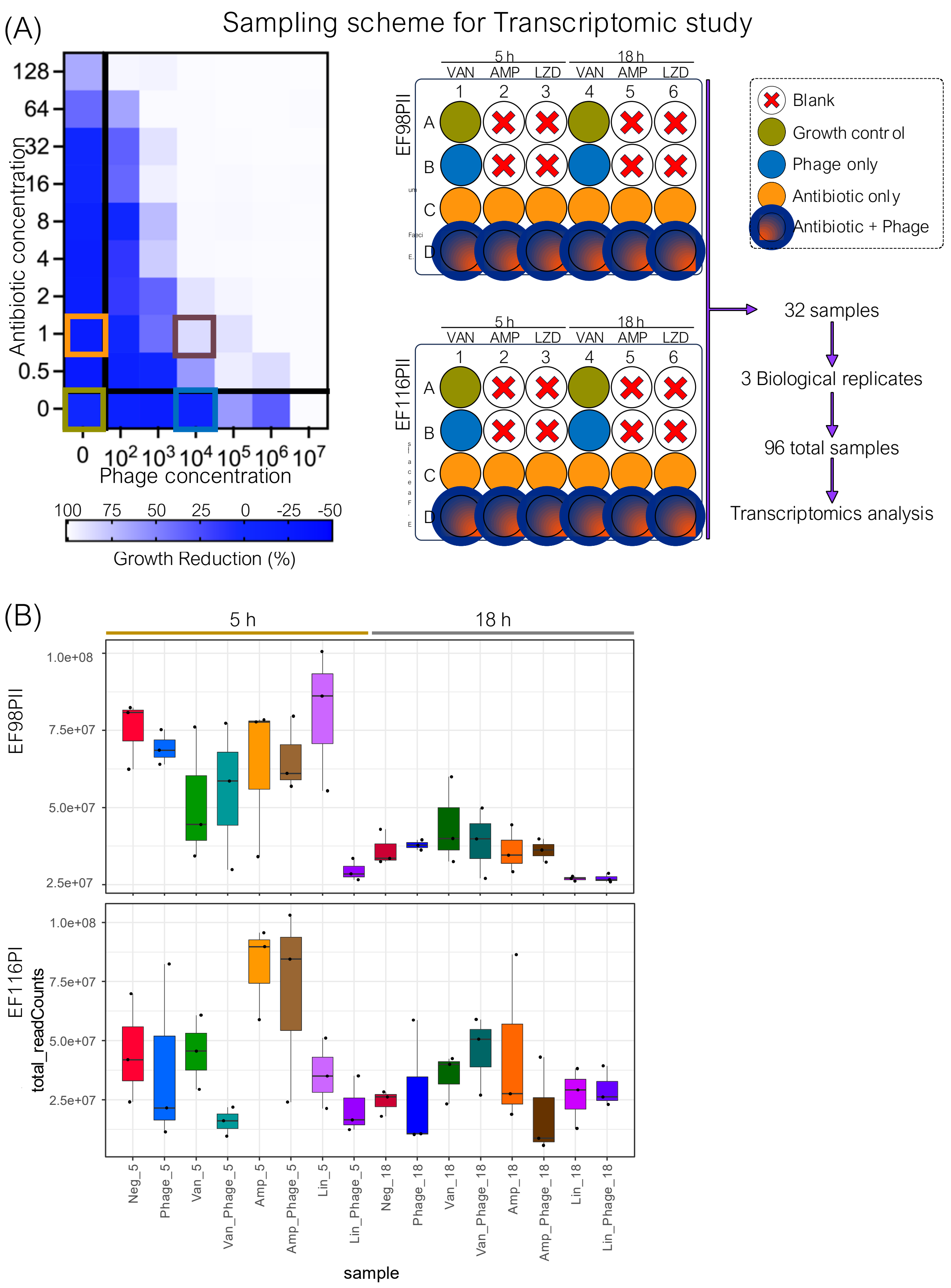

Figure S4. Transcriptomic analysis of phage-antibiotic synergy. (A) Overview of cell sampling scheme for transcriptomic analysis of phage-antibiotic synergy conditions. The best phage-antibiotic pair for each antibiotic with single phage against one bacterial strain for each species was selected and co-culture was repeated along with growth and phage only controls to harvest bacterial cells to perform total RNA extraction. (B) Boxplot shows Interquartile Range (IQR) which depicts the distribution of total read counts. The bottom and top lines of the boxes indicate the first and third quartiles respectively with, the center line marking the median. Whiskers represent the smallest (y-min) and largest (y-max) observations within 1.5 times the IQR from the first and third quartiles respectively. Individual datapoints are indicated by the black dots.

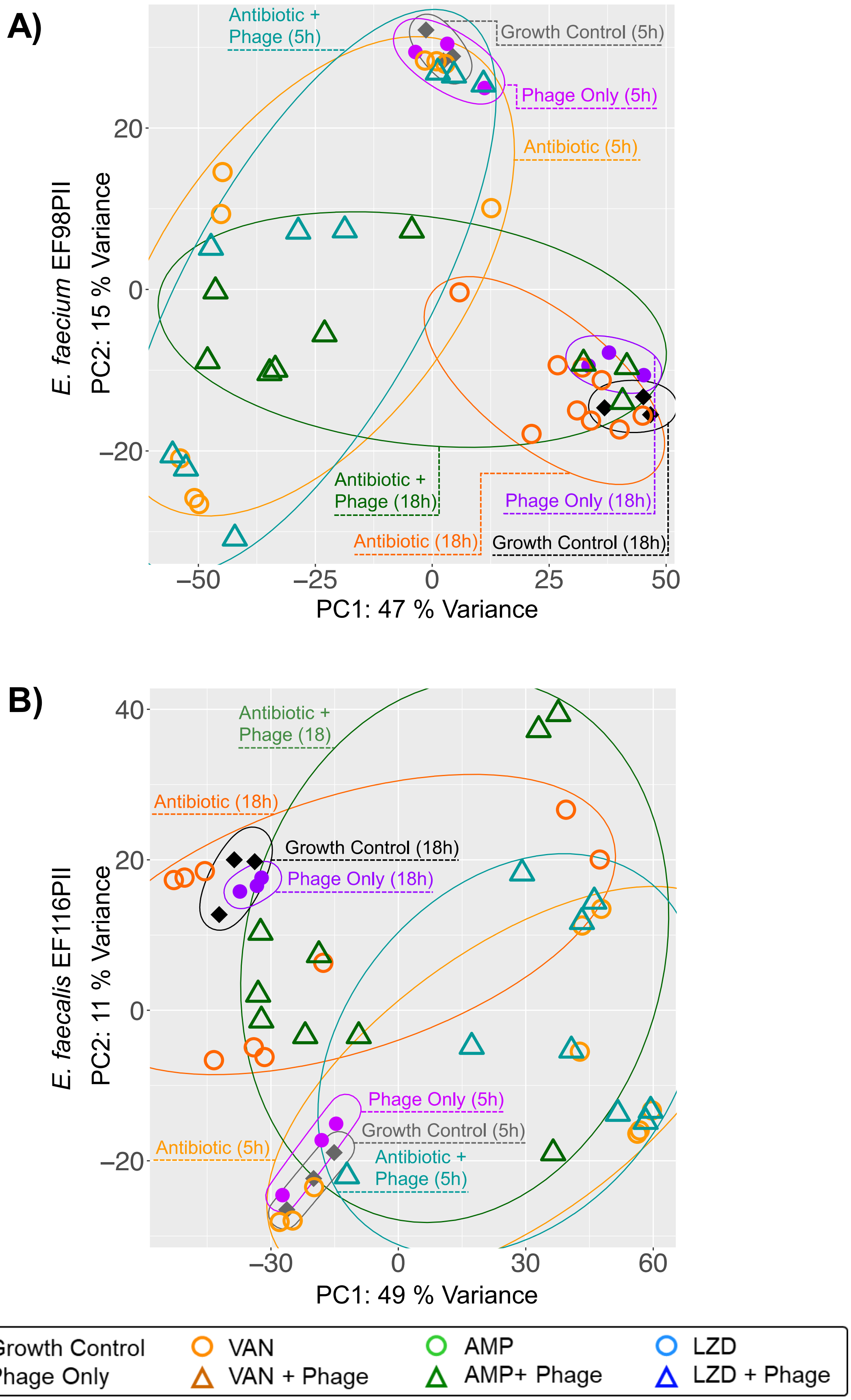

Figure S5. Principal component analysis (PCA) showing clustering of samples by treatment and time. Scatter plots of the first two principal components of the normalized gene expression profile of all the samples. The ellipse encircles the three biological replicates for individual experimental conditions and were drawn at tolerance cutoff of 0.01.
